# Supplementary material for: Development of a computer-aided design software for the quantitative evaluation of aesthetic damage
Source: PLoS One. 2019 Dec 18;14(12):e0226322. doi: 10.1371/journal.pone.0226322 (PMC6919621; doi:10.1371/journal.pone.0226322)
Supplement: S1 Code site — 1. (ZIP) [file pone.0226322.s001.zip › [EESC_jr][Projeto]Relatorio_07_21/vendors/select2/docs/_includes/nav/options-old.html]

- Core Options
  - Declaring configuration in the `data-*` attributes
  - AMD compatibility
  - Displaying selections
  - Returning and displaying results
- Dropdown
  - Attached to body
  - Attached below the container
  - Search
  - Select the highlighted option on close
  - Close the dropdown when a result is selected
- Events
  - Public events
  - Internal events
- The plugin system (adapters)
  - All adapters
  - Container (selection)
  - Data set
  - Dropdown
  - Results
- Setting default options
- Backwards compatibility
  - Simplified function for matching data objects
  - Old initial selections with `initSelection`
  - Querying old data with `query`
  - Compatibility with `<input type="text" />`

Back to top
